# Exopolysaccharides from *Lactiplantibacillus plantarum* WLPL04 Alleviate Hyperuricemia by Regulating Uric Acid Metabolism and Gut Microbiota

**DOI:** 10.3390/foods15071206

**Published:** 2026-04-02

**Authors:** Min Wei, Yingsheng Hu, Xiaoxian Li, Xingyi Long, Zhihong Zhang, Xueying Tao, Hua Wei

**Affiliations:** State Key Laboratory of Food Science and Resources, Nanchang University, Nanchang 330047, China

**Keywords:** *Lactiplantibacillus plantarum*, exopolysaccharide, hyperuricemia, uric acid metabolism, gut microbiota

## Abstract

Background: Hyperuricemia (HUA) is associated with excessive uric acid (UA) production, impaired renal excretion, and gut microbial dysbiosis. This study aims to systematically evaluate the alleviating effects of exopolysaccharide (EPS04) derived from *Lactiplantibacillus plantarum* WLPL04 on HUA. Methods: The study employed both in vitro HK-2 cells and in vivo animal studies in HUA mice. Key methods included assessing xanthine oxidase (XOD) activity and reactive oxygen species (ROS) production in vitro and measuring serum UA levels, renal function parameters, XOD activity, and gene expression in vivo. Additionally, 16S rRNA sequencing was used to analyze gut microbiota composition. Results: EPS04 reduced UA production in HK-2 cells by inhibiting XOD activity and downregulating its gene expression while also decreasing XOD-derived ROS. EPS04 significantly lowered serum UA levels and attenuated renal injury in HUA mice. Hepatic *XOD* expression and activity were downregulated, reducing UA production, while UA excretion was enhanced through upregulation of renal *ABCG2* expression. Furthermore, EPS04 increased gut microbiota α-diversity, restored the *Bacteroidota*/*Firmicutes* ratio, and enriched beneficial taxa, including *Akkermansia* and *Dubosiella*. Conclusions: EPS04 alleviates HUA through inhibition of XOD activity, upregulation of renal *ABCG2* expression, and modulation of gut microbiota, suggesting its potential as a nutraceutical biopolymer for dietary management of HUA and related metabolic disorders.

## 1. Introduction

Hyperuricemia (HUA), characterized by persistently elevated serum uric acid (UA) concentrations exceeding physiological thresholds [1], has emerged as a critical global public health issue. With socioeconomic development, lifestyle changes, and shifts in dietary patterns, HUA has been increasingly recognized as the second most prevalent metabolic disorder after diabetes [2]. In addition to its causal role in gout, persistent HUA is strongly associated with hypertension, chronic kidney disease, and metabolic syndrome [3,4]. UA is the end product of purine catabolism in humans, and its production is largely catalyzed by the molybdenum-containing enzyme xanthine oxidase (XOD), which oxidizes hypoxanthine to xanthine and subsequently to UA [5]. Accordingly, current clinical management of HUA mainly relies on XOD inhibitors, such as allopurinol [6] and febuxostat [7]. Although these therapies effectively lower serum UA levels, their long-term use is frequently limited by serious adverse reactions, including rash, hypersensitivity, hepatic dysfunction, renal impairment, and other safety concerns [8,9]. Therefore, there is an urgent need to explore safer and more effective strategies for the prevention and management of HUA.

In recent years, natural bioactive compounds have demonstrated significant potential in reducing UA levels and inhibiting XOD activity. A previous study reported that the brownin from black tea significantly decreased serum UA level by inhibiting hepatic XOD activity [10]. Similarly, polysaccharides from *Polygonatum sibiricum* reduced serum UA level in HUA mice by inhibiting XOD and adenosine deaminase (ADA) activities [11]. Furthermore, caffeoylquinic acids [12] and quercetin [13] have been demonstrated to inhibit XOD through binding to the flavin adenine dinucleotide domain of the enzyme.

Exopolysaccharides (EPS) produced by lactic acid bacteria (LAB) are natural biopolymers with a wide range of biological activities, including antioxidant [14], immunoregulatory [15], and other health-promoting effects. *Lactiplantibacillus plantarum* WLPL04, originally isolated from human breast milk [16], has previously been shown to ameliorate HUA in mice through attenuation of inflammation, modulation of renal urate transporters, and gut microbiota [17]. Notably, *L. plantarum* WLPL04 is also a high-yield EPS producer, generating EPS at a concentration of 426.73 ± 65.56 mg/L after 24 h of fermentation, and its EPS04 is a heteropolysaccharide with a molecular weight of 6.61 × 10^4^ Da composed of xylose, glucose, and galactose at an approximate molar ratio of 3.4:1.8:1 [18]. EPS04 has been reported to exhibit antioxidant activity in vitro and in a Caco-2 cell model, significantly reducing reactive oxygen species (ROS) production [19]. Moreover, *L*. *plantarum* can adhere to Caco-2 epithelial cells, which may enhance its interaction with the host intestinal mucosa through surface proteins and polysaccharides.

However, it remains unclear whether EPS04 directly contributes to the alleviation of HUA, what mechanisms may underlie its effects, and whether its regulatory role differs from that of the parent strain *L. plantarum* WLPL04. Based on these observations, we hypothesized that EPS04 may exert anti-hyperuricemia effects, which were systematically evaluated in HK-2 cells and a HUA mouse model.

## 2. Materials and Methods

### 2.1. Strain Culture, EPS Isolation, and Purification

Previously obtained from human breast milk, the EPS-producing strain *L. plantarum* WLPL04 was cultured in anaerobic de Man, Rogosa, and Sharpe (MRS) broth (Beijing Solarbio Science and Technology Co. Ltd., Beijing, China) at 37 °C [16]. With a few minor adjustments, EPS04 was extracted and purified as previously described, and its structural characteristics have been described in detail [18]. Briefly, the supernatant was obtained by centrifuging the culture to remove bacterial cells (9000× *g*, 5 min). After adding three volumes of pre-cooled absolute ethanol, the mixture was kept at 4 °C for 48 h, followed by centrifugation at 10,000× *g* for 20 min at 4 °C. The precipitate was dialyzed (MWCO: 8000–14,000 Da) for 3 days at 4 °C after being resuspended in Milli-Q water (Millipore, Shanghai, China). After treating the crude EPS with DNase I (2.5 μg/mL) and protease E (50 μg/mL), trichloroacetic acid was added to a final concentration of 12% to precipitate residual enzymes and peptides. The mixture was centrifuged at 10,000× *g* for 20 min at 4 °C, and the supernatant was adjusted to pH 4.0 to 5.0 with 10 M NaOH. The solution was then dialyzed against Milli-Q water for 48 h at 4 °C and lyophilized.

### 2.2. HK-2 Cell Model

**Figure 1:**
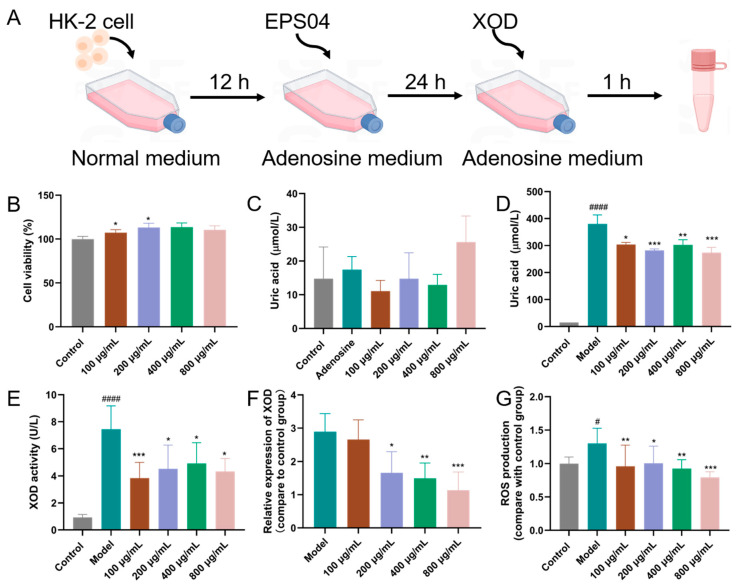
Effect of EPS04 on adenosine and XOD-induced hyperuricemia in HK-2 cells. (**A**) Schematic illustration of the in vitro experimental workflow. (**B**) Cell viability of HK-2 cells treated with EPS04 (100–800 μg/mL). (**C**) UA levels in culture supernatants after adenosine stimulation with EPS04 (100–800 μg/mL), in the absence of exogenous XOD. (**D**) UA level in adenosine-treated HK-2 cells after XOD addition. (**E**) XOD activity in cell culture supernatants. (**F**) Relative expression level of XOD in HK-2 cells. (**G**) ROS production in HK-2 cells. Data are presented as mean ± SD (n = 3 independent experiments). # *p* < 0.05, #### *p* < 0.0001 vs. Control group; * *p* < 0.05, ** *p* < 0.01, *** *p* < 0.001 vs. Model group. Control, untreated cells; Model, adenosine + XOD–treated cells without EPS04.

Human kidney proximal tubular epithelial cells (HK-2) were obtained from the Institute of Cell Biology, Chinese Academy of Sciences (Shanghai, China). Cells were cultured in RPMI 1640 medium with 10% fetal bovine serum at 37 °C in a humidified atmosphere supplemented with 5% CO_2_. Under standard culture conditions, HK-2 cells were seeded at a density of 1 × 10^5^ cells/well into 6-well plates and allowed to stabilize for 12 h. The medium was replaced with RPMI 1640 medium containing adenosine (0.5 mmol/L) and varying concentrations of EPS04, and the cells were incubated for 24 h. Subsequently, XOD (0.005 U/mL) was then added directly and incubated for 1 h. At the end of the reaction, both culture supernatants and cell pellets were collected for subsequent analysis. The overall experimental workflow is illustrated in Figure 1A. The CCK-8 assay kit (Beyotime Biotechnology, Shanghai, China) was used to measure cell viability according to the manufacturer’s protocol. Supernatant UA level and XOD activity were determined using commercial assay kits (Nanjing Jiancheng Bioengineering Institute, Nanjing, China). Intracellular ROS were quantified with a fluorescent probe-based ROS detection kit (Beyotime Biotechnology, Shanghai, China) following the manufacturer’s instructions.

### 2.3. Animal Procedure

**Figure 2:**
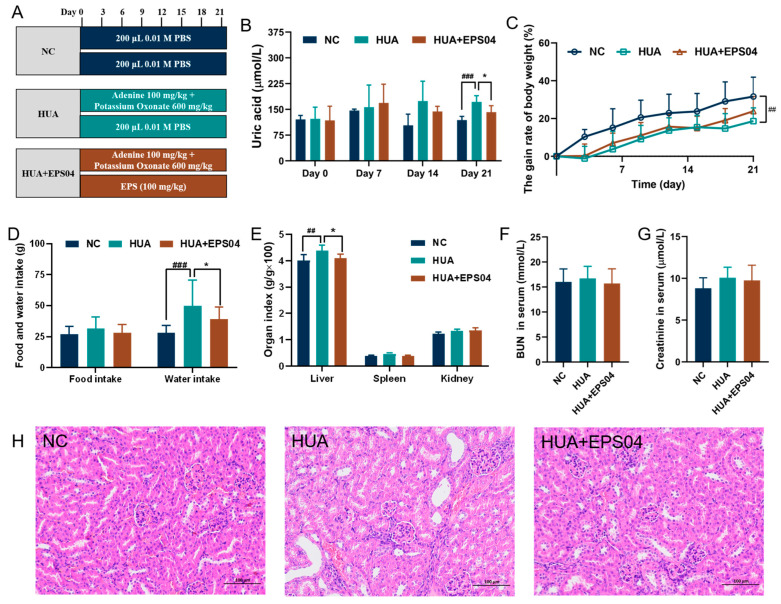
EPS04 ameliorated hyperuricemia and associated renal impairment in mice. (**A**) Animal experimental design of the HUA model and EPS04 intervention. (**B**) Serum UA levels measured at days 0, 7, 14, and 21. (**C**) Body weight gain rate during the 21-day experimental period. (**D**) Food and water intake. (**E**) Organ indices (organ weight/body weight, g/100 g) of liver, spleen, and kidney. (**F**) Serum BUN levels. (**G**) Serum creatinine levels. (**H**) Representative H&E staining images of kidney tissues (scale bar = 100 μm). Data are presented as mean ± SD (n = 6–7 mice per group). ## *p* < 0.01, ### *p* < 0.001 vs. NC; * *p* < 0.05 vs. HUA group. NC, normal control; HUA, hyperuricemia model; HUA+EPS04, hyperuricemia model treated with EPS04.

C57BL/6 male mice (18–20 g) were purchased from Wuhan Myhalic Biotechnology Co., Ltd. (Wuhan, China). All the mice used in this investigation were acclimated to a standard diet and fresh water under a 12 h light/dark cycle at a temperature of 23 ± 1 °C and a humidity of 54 ± 2% for 1 week. The protocol was approved by Wuhan Myhalic Biotechnology Co., Ltd. Animal Experimentation Ethics Committee (HLK-20240617-002). C57BL/6 mice were randomly divided into three groups, each consisting of seven mice: normal group (NC), model group (HUA), and EPS04 group (HUA+EPS04), as shown in Figure 2A. Except for the NC, mice in other groups were established as an HUA model by intragastrical administration with potassium oxonate (600 mg/kg) and adenine (100 mg/kg) every three days (days 0, 3, 6, 9, 12, 15, 18, and 21). Simultaneously, EPS04 (100 mg/kg) was administered daily by oral gavage from day 0 to day 21, starting 2 h after HUA model induction. On day 21, mice were euthanized, and liver, spleen, kidneys, and cecal contents were collected and stored at −80 °C for subsequent analysis. And one of the kidney tissues was placed in 4% paraformaldehyde fixative for histological analysis and staining. The blood samples were clotted for 1 h at room temperature and then centrifuged (3000× *g* for 4 min) to separate the serum for the biochemical measurements.

### 2.4. Urine Collection and Analysis

On day 21, urine samples were collected from each mouse using individual metabolic cages (one mouse per cage) throughout a 12 h period without food but with free access to water, and sterile tubes were used to collect urine. To remove debris, urine samples were centrifuged at 4 °C, 3000× *g* for 10 min, and the supernatants were stored at −80 °C until biochemical analysis. Frozen urine samples were thawed in a 60 °C water bath and diluted tenfold with distilled water. UA level was quantified using commercial assay kits following the manufacturers’ protocols (Nanjing Jiancheng Bioengineering Institute, Nanjing, China).

### 2.5. Biochemical Index Assay

Before analysis, the serum samples that had been kept at ultra-low temperature were thawed at room temperature. Levels of UA, blood urea nitrogen (BUN), and creatinine in serum were quantified using commercial assay kits following the manufacturers’ protocols (Nanjing Jiancheng Bioengineering Institute, Nanjing, China). For hepatic enzyme measurements, liver tissue was homogenized in ice-cold saline at a ratio of 1:9 (*w*/*v*) to provide a 10% homogenate, which was then centrifuged at 3000× *g* for 10 min to obtain the clarified supernatant. Hepatic XOD and ADA activities were subsequently assayed using the corresponding kits from the same supplier, strictly adhering to the provided instructions.

### 2.6. RT-qPCR Analysis

Total RNA was extracted using TRIzol reagent, and cDNA was synthesized using the PrimeScript^TM^ RT reagent kit with gDNA Eraser (Takara Biomedical Technology, Beijing, China) according to the manufacturer’s instructions. Quantitative PCR (qPCR) was conducted using the SYBR^®^ Premix Ex Taq II kit (Takara Biomedical Technology, Beijing, China) on an ABI 7900 HT fast real-time PCR system (Applied Biosystems, Foster City, CA, USA). The transcriptional levels of *XOD*, *ABCG2*, *URAT1*, and *GLUT9* in mice and HK-2 cells were analyzed. The qPCR conditions were as follows: 95 °C for 5 min, followed by 40 cycles of 95 °C for 30 s, 56 °C for 30 s, and 72 °C for 30 s. Reactions were run in triplicate, and data were analyzed using the 2^−∆∆Ct^ method, with β-actin gene as the reference gene. Primers sequences are listed in Table 1.

### 2.7. Histopathological Evaluation

Mice were euthanized, and the kidneys were excised and washed with saline solution. The right kidney was then preserved in 4% paraformaldehyde and fixed for 48 h at room temperature. The kidney was then dehydrated, defatted in xylene, embedded in paraffin, and sliced into sections that were 5 μm thick. A light microscope (NIKON digital sight DS-FI2, Tokyo, Japan) was used to take and analyze the images.

### 2.8. Bacterial DNA Extraction and Sequence Data Analysis

Total genomic DNA was isolated from cecal contents samples using the QIAamp DNA Isolation Kit (Qiagen, Hilden, Germany) according to the manufacturer’s protocol. DNA concentration was determined by NanoDrop (Thermo Fisher Scientific, Waltham, MA, USA). The V3 + V4 region of the 16S rDNA was amplified using specific primers with a barcode. The primer sequences were 341F: CCTACGGGNGGCWGCAG and 806R: GGACTACHVGGGTATCTAAT. The purified amplification products were ligated to sequencing junctions to construct libraries and sequenced on an Illumina platform. Raw sequencing reads were processed using the QIIME2 pipeline. Sequences were demultiplexed, quality filtered, denoised, and chimera-checked using the DADA2 algorithm to generate amplicon sequence variants (ASVs). Species annotation, microbial composition analysis, indicator species analysis, α-diversity and β-diversity analyses, as well as other downstream analyses, were performed based on sequence and abundance data at the ASV level.

### 2.9. Statistical Analysis

All experimental data are shown as the mean ± standard deviation (SD). Statistical analysis was performed using one-way ANOVA followed by Tukey’s multiple comparisons test for comparisons among multiple groups. For comparisons between two groups only, an unpaired two-tailed Student’s *t*-test was used. All statistical analyses were carried out using GraphPad Prism v10.4.2 (GraphPad Software, Inc, La Jolla, CA, USA). Statistical significance was denoted as *p* < 0.05, and all experiments were replicated three times.

## 3. Results

### 3.1. Effect of EPS04 on Hyperuricemia HK-2 Cells Induced by Adenosine and XOD

The schematic illustration of the EPS04 intervention in HUA HK-2 cells is shown in Figure 1A. EPS04 at concentrations ranging from 100 to 800 μg/mL exhibited no cytotoxic effect on HK-2 cells (Figure 1B). Without exogenous XOD supplement, there was no significant difference in UA level among all groups. In contrast, with the addition of XOD, the UA level increased up to 380.15 ± 33.19 μmol/L in the model group but significantly decreased with EPS04 intervention; correspondingly, the XOD activity in the supernatant presented the same tendency. At the mRNA level, EPS04 intervention at concentrations ≥ 200 μg/mL significantly downregulated XOD expression in HK-2 cells. Furthermore, the pronounced increase in intracellular ROS was effectively mitigated by EPS04 (Figure 1G), demonstrating that XOD-associated oxidative stress was markedly alleviated.

### 3.2. Effect of EPS04 on Physiological in HUA Mice

Based on the inhibition tendency of UA production and XOD activity observed in vitro, EPS04 was further evaluated for its in vivo intervention effect, as described in Figure 2A. The HUA model was successfully established in mice, as evidenced by a significant elevation in serum UA level on day 21 (171.7 ± 17.9 μmol/L, *p* < 0.001), whereas it was reduced to 141.9 ± 18.7 μmol/L after EPS04 intervention (*p* < 0.05, Figure 2B). The significant body weight loss induced by HUA (*p* < 0.01, Figure 2C) was greatly alleviated by EPS04 intervention (Figure 2C), and water intake presented the same tendency (*p* < 0.05, Figure 2D). For organ indexes, the liver index was significantly reduced by EPS04 intervention (*p* < 0.05, Figure 2E). Although EPS04 treatment did not affect serum BUN (Figure 2F) or creatinine (Figure 2G) levels, renal pathological lesions were greatly alleviated (Figure 2H).

### 3.3. Effect of EPS04 on UA Metabolism and Transport Regulation

**Figure 3:**
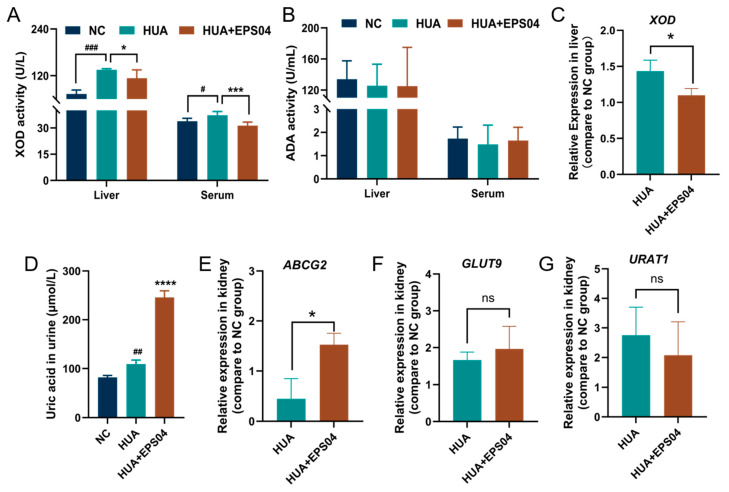
EPS04 modulated uric acid production and excretion pathways in hyperuricemic mice. (**A**) XOD activity in the liver and serum. (**B**) ADA activity in the liver and serum. (**C**) Hepatic mRNA expression of *XOD*. (**D**) Urinary uric acid levels. (**E**–**G**) Renal mRNA expression of *ABCG2*, *GLUT9*, and *URAT1*. # *p* < 0.05, ## *p* < 0.01, ### *p* < 0.001 vs. NC; * *p* < 0.05, *** *p* < 0.001, **** *p* < 0.0001 vs. HUA group. NC, normal control; HUA, hyperuricemia model; HUA+EPS04, hyperuricemia model treated with EPS04.

To further elucidate the mechanisms underlying UA reduction by EPS04, the activity and expression of key enzymes and transporters associated with UA metabolism were investigated. The EPS04 intervention significantly inhibited XOD activity but not ADA activity in both liver (*p* < 0.05) and serum (*p* < 0.001) (Figure 3A,B), which was accompanied by a marked downregulation of hepatic *XOD* expression (*p* < 0.05, Figure 3C). Meanwhile, urinary UA level was significantly increased after EPS04 intervention (*p* < 0.0001, Figure 3D). Consistently, EPS04 significantly upregulated the expression of the urate efflux transporter *ABCG2* in the kidney (*p* < 0.05, Figure 3E), while the expression of renal urate reabsorption-related transporters *GLUT9* and *URAT1* remained unchanged (Figure 3F,G). These findings indicated that the UA-lowering effect of EPS04 was mainly mediated by reducing hepatic UA synthesis and promoting UA excretion, rather than by regulating the expression of renal UA reabsorption.

### 3.4. Effect of EPS04 on Gut Microbes in Hyperuricemia Mice

**Figure 4:**
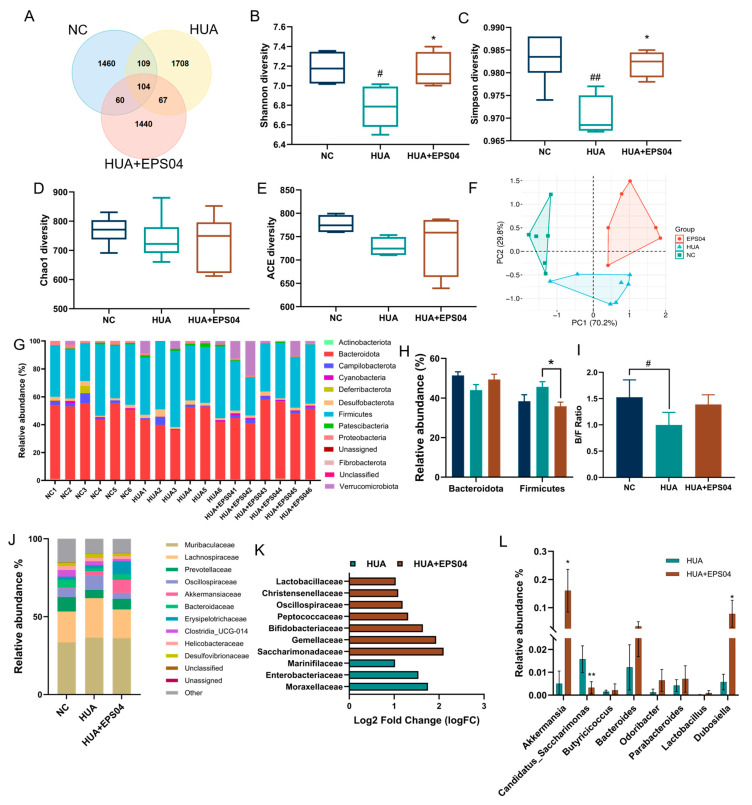
EPS04 regulated the gut microbiota composition in hyperuricemia mice. (**A**) Venn diagram of OTUs among groups, with different colors representing different groups. (**B**–**E**) α-diversity indices, including Shannon, Simpson, Chao1, and ACE. (**F**) Principal coordinate analysis (PCoA) based on Bray–Curtis distance. (**G**) Relative abundance of gut microbiota at the phylum level in every sample (top 10). (**H**) Relative abundance of *Bacteroidota* and *Firmicutes*. (**I**) B/F ratio. (**J**) Relative abundance of gut microbiota at the family level among groups (top 10). (**K**) Differentially enriched bacteria between HUA and HUA+EPS04 groups (log2 fold change) at the family level. (**L**) Relative abundance plots displaying the differences in the general microbial community at the genus level. # *p* < 0.05, ## *p* < 0.01 vs. NC; * *p* < 0.05, ** *p* < 0.01 vs. HUA group. NC, normal control; HUA, hyperuricemia model; HUA+EPS04, hyperuricemia model treated with EPS04.

The gut microbiota is closely associated with UA metabolism. Venn analysis (Figure 4A) showed that only 104 OTUs were shared among all groups, indicating pronounced differences in gut microbial composition between the HUA and EPS04 groups compared with the NC. EPS04 treatment significantly increased microbial diversity, as reflected by higher Shannon and Simpson indices, while richness indices (Chao1 and ACE) remained unchanged (Figure 4B,E). Principal coordinates analysis (PCoA) further demonstrated a clear separation of microbial communities among the three groups (Figure 4F). At the phylum level, the HUA group exhibited a decreased relative abundance of *Bacteroidota* and an increase in *Firmicutes* (Figure 4G,H), resulting in a significantly lower *Bacteroidota*/*Firmicutes* (B/F) ratio compared with the NC (*p* < 0.05, Figure 4I). After EPS04 intervention, *Firmicutes* abundance was significantly decreased (*p* < 0.05, Figure 4G,H) while *Bacteroidota* abundance increased, and therefore restored the B/F ratio toward normal levels. At the family level, the relative abundance of *Prevotellaceae* and *Bacteroidaceae* was reduced in the HUA compared with the NC (from 9.16% to 5.11% and from 4.76% to 1.73%, respectively, Figure 4J). Following EPS04 treatment, the abundance of *Bacteroidaceae* and *Erysipelotrichaceae* increased to 3.35% and 8.28%, respectively (Figure 4J). Differential abundance analysis further showed that *Lactobacillaceae*, *Christensenellaceae,* and *Bifidobacteriaceae* were significantly enriched after EPS04 intervention, while potentially detrimental taxa such as *Enterobacteriaceae* and *Moraxellaceae* were enriched in the HUA group (Figure 4K). At the genus level, beneficial taxa, including *Akkermansia* and *Dubosiella* (*p* < 0.05), were significantly increased, whereas the relative abundance of *Candidatus_Sacchairmonas* was reduced after EPS04 intervention (*p* < 0.01, Figure 4L). Collectively, these results demonstrated that EPS04 effectively modulated gut microbiota structure and alleviated HUA-associated dysbiosis.

## 4. Discussion

Accumulating evidence suggests that LAB represent a promising nutritional strategy for the prevention of HUA [20,21]. EPS derived from LAB possess prebiotic properties, including antioxidant activity and the ability to attenuate ROS production [19]; however, their potential role in ameliorating HUA has not been well elucidated. In the present study, the alleviation of HUA by EPS04 was associated with reducing UA production by inhibiting XOD activity, enhancing urate elimination via upregulation of the efflux transporter *ABCG2* in the kidney, and reshaping gut microbiota in HUA mice.

HUA arises from an imbalance between UA overproduction and insufficient excretion. One of the major contributors to UA overproduction is increased XOD catalytic activity, which not only promotes UA generation but is also accompanied by increased production of ROS [22,23]. This process further aggravates metabolic stress, leading to cellular damage and inflammatory responses [1]. In the present study, EPS04 was shown to suppress XOD activity and downregulate *XOD* transcription both in vitro and in vivo, suggesting that suppression of XOD-related UA production may contribute to its anti-hyperuricemia effect. However, the mode of XOD inhibition remains unclear, as it may involve competitive, non-competitive, uncompetitive, or mixed inhibition and may also be associated with the structural characteristics of EPS04, which requires further validation by enzyme kinetic analysis and studies on the relationship between structure and activity. In addition, the EPS04 intervention significantly reduced ROS production in HK-2 cells, indicating its capacity to alleviate XOD-associated oxidative stress at the cellular level. Similarly, polysaccharides derived from *Polygonatum sibiricum* have been reported to effectively decrease serum UA levels by inhibiting XOD activity and suppressing ROS production in HK-2 cells [11].

Urate homeostasis is critically determined by the balance between renal and intestinal excretion and tubular reabsorption [24], a process regulated by multiple urate transporters, including *ABCG2*, *URAT1*, and *GLUT9*. In the present study, the EPS04 intervention significantly promoted the excretion of UA in urine by 2.25-fold compared with the HUA group. Since ABCG2 is a key urate efflux transporter abundantly expressed on the apical membrane of renal proximal tubules [25], it plays a central role in renal urate secretion. Consistently, EPS04 selectively upregulated the expression of urate efflux transporter *ABCG2* by 3.41-fold, while the expression levels of the urate reabsorption-related transporters *URAT1* and *GLUT9* remained unchanged compared with the HUA group. These findings indicated that transcriptional response to EPS04 differs from that observed for *L. plantarum* WLPL04, which has been shown to downregulate *URAT1* and *GLUT9* while upregulating *ABCG2* [17]. This divergence suggests that EPS04 represents only one functional component of the probiotic’s anti-hyperuricemia activity. Live bacteria may additionally regulate reabsorption-related pathways through direct purine utilization and metabolite production. Moreover, the lack of significant changes in *URAT1* and *GLUT9*, together with the absence of significant changes in BUN and creatinine, suggests that the renal effects of EPS04 may be selective and that comprehensive renal protection was not fully demonstrated under the present experimental conditions. Taken together, the serum UA-lowering effect of EPS04 might be mediated by reducing hepatic UA synthesis and promoting urinary UA excretion through upregulation of *ABCG2* expression. These findings were consistent with previous reports showing that chicory extract [26] and tuberindine A [27] alleviate HUA by enhancing *ABCG2*-mediated urate excretion.

Gut microbes are increasingly recognized as key contributors to UA homeostasis in HUA, and modulation of the gut microbiota may represent an effective strategy for improving HUA [28]. Our previous study showed that *L. plantarum* WLPL04 intervention elevated the relative abundance of *Bacteroidota* while reducing *Firmicutes* [17], a trend that was similarly observed following EPS04 intervention. Moreover, *L. plantarum* WLPL04 intervention significantly enriched serum UA-negative related taxa (*Akkermansia* and *Dubosiella*) [17]. Notably, EPS04 intervention also markedly increased the abundance of these beneficial genera, with *Akkermansia* and *Dubosiella* enriched by 32.12-fold and 13.74-fold, respectively. *Akkermansia*, a well-recognized mucin-degrading commensal, has been reported to enhance gut barrier function and anti-inflammatory effects [29]. Importantly, *Akkermansia muciniphila* has been reported to lower serum UA, inhibit hepatic XOD, and regulate renal urate transporters (*URAT1*, *GLUT9*, and *ABCG2*) [30]. Collectively, EPS04 appears to function as a key microbiota-modulating effector that mediates, at least in part, the anti-hyperuricemia effects of *L. plantarum* WLPL04, but its causal contribution remains to be further verified.

While the present study supports the anti-hyperuricemic potential of EPS04, further work is needed to clarify its mechanism and application value. Future studies should include enzyme kinetics, transporter functional assays, causal microbiota validation, and appropriate comparator treatments. Larger, longer-term, multi-dose studies in both sexes are also needed. In addition, further structural characterization and bioactivity validation will help elucidate the structure–function relationship of EPS04.

## 5. Conclusions

EPS04 ameliorates HUA in mice through a multifaceted mechanism involving the regulation of UA metabolism and gut microbiota homeostasis. EPS04 suppresses UA production by inhibiting XOD activity while concurrently enhancing urate elimination through the selective upregulation of the efflux transporter *ABCG2* in the kidney. Moreover, EPS04 reshapes the gut microbiota by enriching beneficial taxa, including *Akkermansia*. As a novel, high-yield EPS derived from *L. plantarum* WLPL04 isolated from human breast milk, EPS04 shows promise as a functional food ingredient or dietary intervention for the nutritional management of HUA and related metabolic disorders.

## Figures and Tables

**Table 1:** Primers used for qPCR.

Genes	Forward Primer (5′-3′)	Reverse Primer (3′-5′)
*m-XOD*	ATGACGAGGACAACGGTAGAT	TCATACTTGGAGATCATCACGGT
*m-ABCG2*	GGCCTGGACAAAGTAGCAGA	GTTGTGGGCTCATCCAGGAA
*m-GLUT9*	TGGACTCAATGCGATCTGG	AGAGAAGATAGCAGCCAGTGTTT
*m-URAT1*	CCGCTTCCGACAACCTCA	CTTCTGCGCCCAAACCTATC
*m-β-actin*	GCTCCTCCTGAGCGCAAGTA	CAGCTCAGTAACAGTCCGCC
*h-XOD*	TCTTCCTGGCTGCTTCTATCTT	TGTTCTGTGGTATGTTCCTCCT
*h-β-actin*	CATCCCCCAAAGTTCACAATT	AGTGGGGTGGCTTTTAGGAT

## Data Availability

The original contributions presented in this study are included in the article. Further inquiries can be directed to the corresponding author.
